# Seroprevalence of coxsackievirus A16 antibody among people of various age groups: a systematic review and meta-analysis

**DOI:** 10.1186/s13690-021-00688-z

**Published:** 2021-09-17

**Authors:** Peng Li, Yan Chen, An Tang, Fan Gao, Jian-Bo Yan

**Affiliations:** 1 Zhoushan Municipal Center for Disease Control and Prevention, No.568 Wengshan Road, Zhoushan City, Zhejiang Province 316021 People’s Republic of China; 2 Department of Ophthalmology, Zhoushan Hospital, Zhoushan City, Zhejiang Province 316021 People’s Republic of China

**Keywords:** **S**eroprevalence, Coxsackievirus A16, Hand, foot and mouth disease(HFMD), Meta-analysis

## Abstract

**Background:**

Coxsackie virus group A type 16 (CoxA16) is the main pathogen and usually an alternative to or joins in prevalence with enterovirus 71 (EV71) causing hand, foot and mouth disease (HFMD). The objective of this study was to estimate the seroprevalence of CoxA16 antibody among people of various age groups by a systematic review and meta-analysis.

**Methods:**

The literature of seroprevalence of CoxA16 antibody among people has been systematically searched through databases from the date of their establishment to Jan. 2021. Estimates of seroprevalence of CoxA16 antibody by gender and age groups have been summarized by using fixed- and random- effect models. All analyses have been conducted in STATA version 12.0 software.

**Results:**

A total of 14 publications with 9 in English and 5 in Chinese containing 9562 samples were finally included in the meta-analysis. The seroprevalence of CoxA16 antibody reported in different studies range from 24.85 to 76.92 %. Meta-analysis has revealed that the seroprevalence of CoxA16 antibody was 56.3 % (95 %CI: 47.7 %~64.9 %) in the overall population and 55.1 % (95 %CI: 44.1 %~66.1 %) in the Chinese population. Subgroup analysis by gender has revealed that the seroprevalence of CoxA16 antibody was 56.1 % (95 %CI: 45.2 %~67.1 %) in males and 60.0 % (95 %CI: 50.0 %~69.9 %) in females. Subgroup analysis by age groups has revealed that the seroprevalence of CoxA16 antibody was 49.1 % (95 %CI: 36.2 %~62.0 %) in the 0 ~ 5 age group and 63.9 % (95 %CI: 53.1 %~74.7 %) in the over 5 age group. Begg’s funnel plots have suggested that there were no publication bias in all groups. Sensitive analysis has suggested that the result of the meta-analysis was stable.

**Conclusions:**

The seroprevalence of CoxA16 antibody was closely related to age. Children under 5 years old were the main susceptible groups for CoxA16 and also the key groups for the prevention and control of HFMD.

**Supplementary Information:**

The online version contains supplementary material available at 10.1186/s13690-021-00688-z.

## Background

HFMD, an infectious disease caused by enterovirus, is common in children under 5 years of age, with the highest morbidity and mortality among children aged from 12 to 23 months [1, 2]. HFMD can cause fever, rashes and ulcers on hands, foot, and mouth. A small number of patients may develop neurological and cardiopulmonary complications such as aseptic meningitis, brainstem encephalitis, acute flaccid paralysis, pulmonary edema, and cerebral hemorrhage, or even death [3]. Viruses causing HFMD belong to the enterovirus family of the small RNA family, among which enterovirus 71 (EV-71) and Coxazivirus A16 (CoxA16) are the most common [4]. CoxA16 can be transmitted by fecal-oral route and close contact, easily causing outbreaks and epidemics in preschool children [5]. Since 1957, when the first HFMD case was reported in Toronto, HFMD has alternated or co-circulated with EV71, causing several pandemics worldwide [4–6]. In the past 20 years, HFMD has been prevalent in the Asia-Pacific region, including Singapore, Malaysia, Japan, South Korea, Thailand, Vietnam and Taiwan of China [7]. On May 2, 2008, HFMD was listed as a notifiable infectious disease in China.

China’s statutory reporting system for infectious diseases features a passive monitoring network, which means under-reporting is inevitable. At the same time, some cases may not be seen in hospitals, so they cannot be captured. The limitations of such a passive monitoring may lead to incomplete reflection of the prevalence of HFMD in China with the existing monitoring data. It has been reported that 50 % ~ 80 % of enterovirus infections are recessive infections with no or mild clinical symptoms, and only a few are manifested as dominant infections [8]. Serological investigation of the antibody levels of EV-A71 and CoxA16 in the population is the most effective way to indirectly reflect the prevalence of HFMD. In the meantime, it can be used to understand the dynamic changes of susceptibilities and immune levels of children at different age, so as to provide reference for the development of vaccination strategies and guide the prevention and control of HFMD.

A previous systematic review and meta-analysis has been conducted to evaluate the seroprevalence of enterovirus 71 antibody among children in China [9]. In this study, we retrospectively retrieved the published epidemiological literature on the seroprevalence of CoxA16 antibody, and the positive rates of CoxA16 antibody in different age groups have been comprehensively analyzed, so as to discuss the susceptibility of the population and the dynamic changes of immune status, and provide reference for the prevention and control of HFMD in the future.

## Materials and methods

### Search strategy

China National Knowledge Infrastructure (CNKI), WanFang Data, PubMed, EMbase, and the Cochrane Library to collect cross-sectional studies on the seroprevalence of CoxA16 antibody among people of various age groups from the date of their establishment to Jan. 2021 have been searched. The following keywords have been used in the literature search: (“hand foot and mouth disease” OR “HFMD” OR “coxsackievirus A16” OR “CA16” OR “CoxA16”) AND (“seroprevalence” OR “seroprevalent” OR “seronegative” OR “seropositive” OR “seroepidemiology” OR “seroepidemiological” OR “serologic” OR “serological” OR “antibody”). In addition, references of included literature have also been retrieved manually to avoid omission of relevant literature in the above-mentioned databases.

### Inclusion and exclusion criteria

The inclusion criteria were as follows: (1) literature on the seroprevalence of CoxA16 antibody among people published by June 2020; (2) type of study: a cross-sectional study that investigated the status of the seroprevalence of CoxA16 antibody; (3) positive rates of CoxA16 antibody can be calculated either explicitly or indirectly in the literature; (4) for repeated studies, the study with the largest sample size was selected. Exclusion criteria were as follows: (1) literature with obvious erroneous data or incomplete data; (2) types of research including review, conference, and other type of literature; (3) literature takes patients with hand foot and mouth disease as subjects.

### Literature screening and data extraction

According to the inclusion criteria and exclusion criteria, two evaluators (PL and YC) have made a preliminary screening by filtering the title and abstract of the literature. After excluding obviously irrelevant literature, full text has been further read to determine the final results for inclusion. Two researchers (PL and YC) have independently screened the literature, extracted and cross-checked the data. In case of differences in the data extraction, the third researcher (AT) assisted in the discussion and decision-making. The missing data have been supplemented by contacting the authors. We have extracted the following information from each eligible article: first author, publication date, survey area, sample size, seroprevalence of CoxA16 antibody, assay method, age range of the studied population, grouping factors.

### Quality assessment

The included studies have been evaluated for bias risk in cross-sectional studies according to 11 evaluation criteria recommended by the Agency for Healthcare Research and Quality (AHRQ), and each item has been answered with “Yes (scored 1 point)”, “No (scored 0 point)” or “Unclear (scored 0 point)” respectively [10]. The full score of quality evaluation is 11 points, with scores ranging from 0 to 3 points, from 4 to 7 points, and from 8 to 11 points representing low quality, medium quality and high quality, respectively.

### Statistical analysis

The study has been conducted following the preferred reporting items for systematic reviews and meta-analysis (PRISMA) statement [11]. The pooled seroprevalence and 95 % confidence interval (CI) was the statistical effect size used to estimate the seroprevalence of CoxA16 antibody among people in different groups. Heterogeneity of the included studies has been determined by Cochran’s Q test and the I^2^ statistic. If the Cochran’s Q test was with *P* < 0.1 and I^2^ ≤ 50 %, indicating that there was no significant difference in the heterogeneity between studies, fixed-effect model was used for meta-analysis [12, 13], otherwise, random-effect model was adopted. Subgroup analysis by gender and age group have also been conducted in the meta-analysis. In addition, the stability of the meta-analysis results has been evaluated in the sensitivity analysis using studies that were excluded one by one. Finally, Begg’s funnel plot and Egger’s linear regression analysis have been used to evaluate publication bias [10]. All statistical analyses have been performed in the STATA 12.0 program (StataCorp LP, College Station, TX, USA). A *P*-value < 0.05 was considered statistically significant.

## Results

### Study search results

**Fig. 1:**
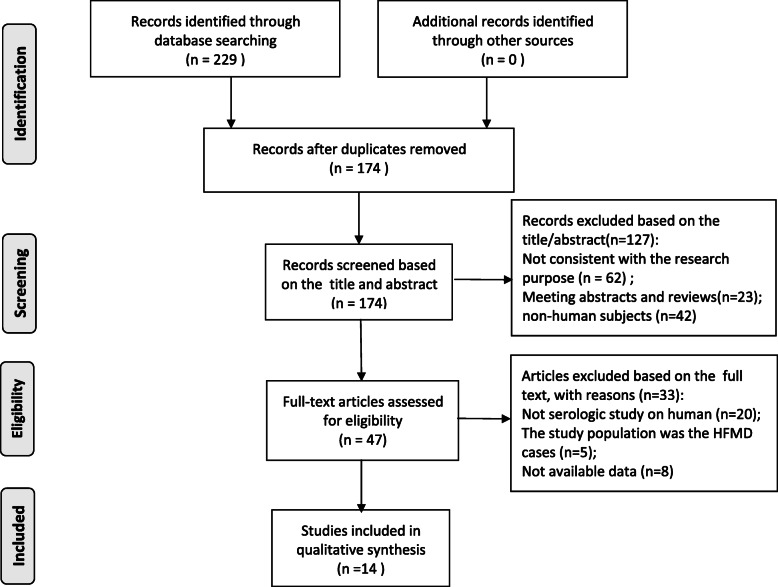
Flow chart of the literature retrieval and selection process

The two authors of this study searched the relevant databases according to the retrieval strategy respectively, and a total of 229 relevant studies have been searched at the initial inspection. The inclusion criteria and exclusion criteria have been used to independently conduct study screening, and the results of data extraction and quality assessment have been compared. After deleting duplicates and screening according to the inclusion and exclusion criteria, a total of 14 studies that met the selection criteria have been included in this meta-analysis [14–27]. A total of 9562 samples have been investigated in 14 studies, among which 5682 had positive neutralizing antibodies against CoxA16, and the seroprevalence of the CoxA16 antibody ranged from 24.85 to 76.92 %. Out of the 14 studies, 5 articles were in Chinese and 9 in English. In addition, 3 medium-quality studies and 11 high-quality studies were included in the studies. The basic information of all studies included in this meta-analysis has been shown in Table 1. The detailed flow chart of article selection for inclusion and exclusion has been presented in Fig. 1.

**Table 1:** Basic information of the studies included in the meta-analysis

First author	Publication year	Country	Location	Sample size	No. positive	Seroprevalence rate (%)	Assay method	Positive threshold	Age range	Group factors	AHRQ scores
Cheng [14]	2016	China	Jiangsu	420	125	29.76	NTA	1:8	0–15 yrs	Age, gender	8
Han [15]	2014	China	Shandong	1000	786	78.60	ELISA	OD450	0–31 yrs	Age, gender	8
Mao [16]	2009	China	Henan	337	123	36.50	NTA	1:8	7–30 mons	Age	7
Song [17]	2020	China	Tianjin	171	107	62.57	NTA	1:8	0–35 yrs	Age, gender	7
Ang [18]	2015	Singapore	Singapore	700	424	60.57	NTA	1:8	1–17 yrs	Age, gender, ethnic	9
Zhu [19]	2012	China	Jiangsu	1530	1024	66.93	NTA	1:8	all ages	Age	7
Lerdsamran [20]	2018	Thailand	Thailand	696	438	62.93	NTA	1:8	6–18 yrs	Age, gender	8
Rabenau [21]	2010	Germany	Germany	1378	989	71.77	NTA	1:8	all ages	Age, gender	8
Wang [22]	2016	China	Shandong	391	230	58.82	NTA	1:8	all ages	Age, gender	9
Wang [23]	2014	China	Jiangsu	715	400	55.94	NTA	1:8	20–50 yrs	Age, gender	9
Li [24]	2013	China	Guangdong	900	390	43.33	NTA	1:8	1–9 yrs	Age, gender	9
Zhu [25]	2010	China	Mixed	515	128	24.85	NTA	1:8	1–5 yrs	Age	8
Zhu [26]	2018	China	Fujian	230	176	76.52	NTA	1:8	all ages	Age, gender	8
Li [27]	2017	China	Tianjin	420	125	29.76	NTA	1:8	all ages	Age, gender	8

### Seroprevalence of CoxA16 antibody

**Fig. 2:**
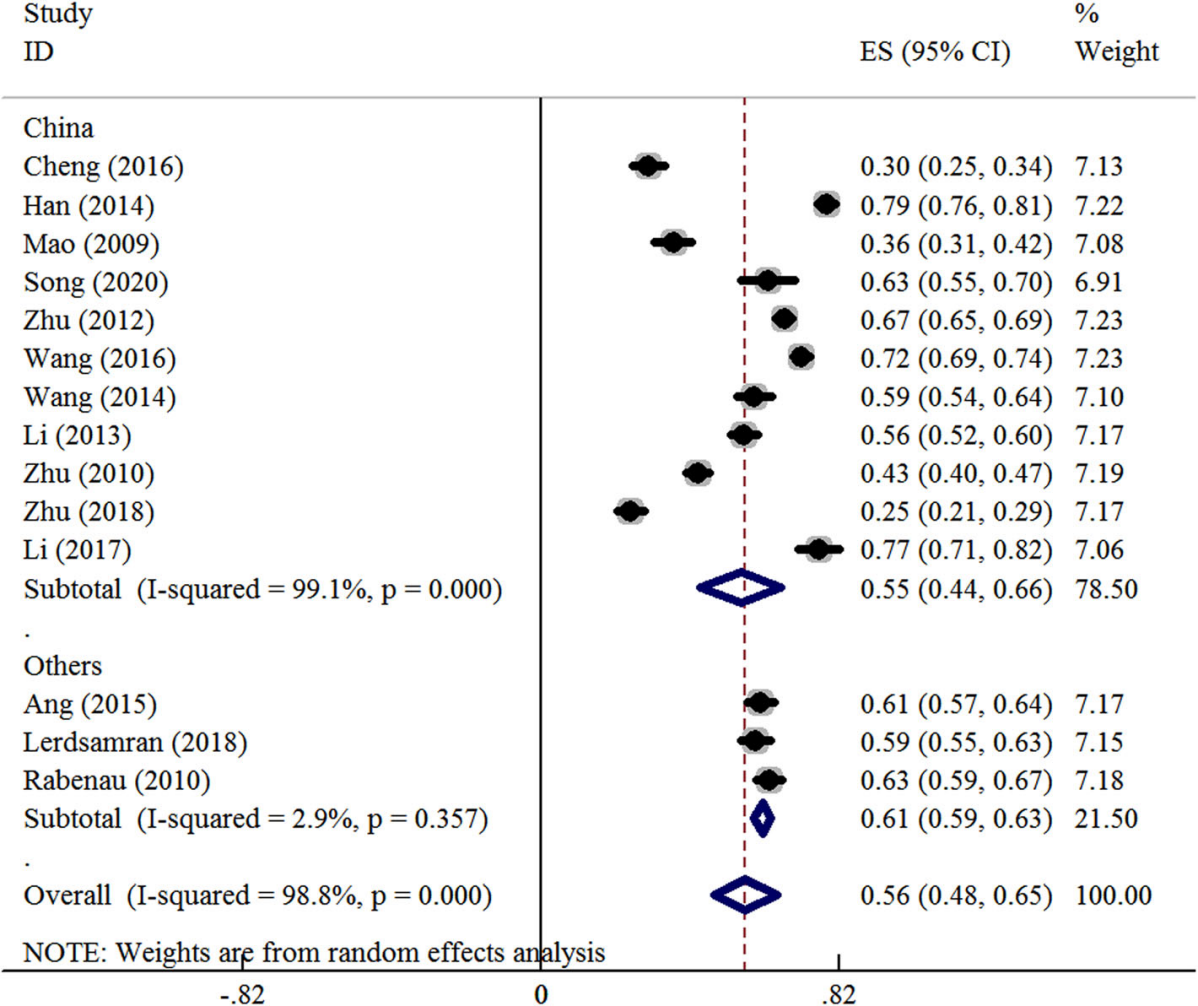
Forest plots for the seroprevalence of CoxA16 antibody among people in the overall population

**Fig. 3:**
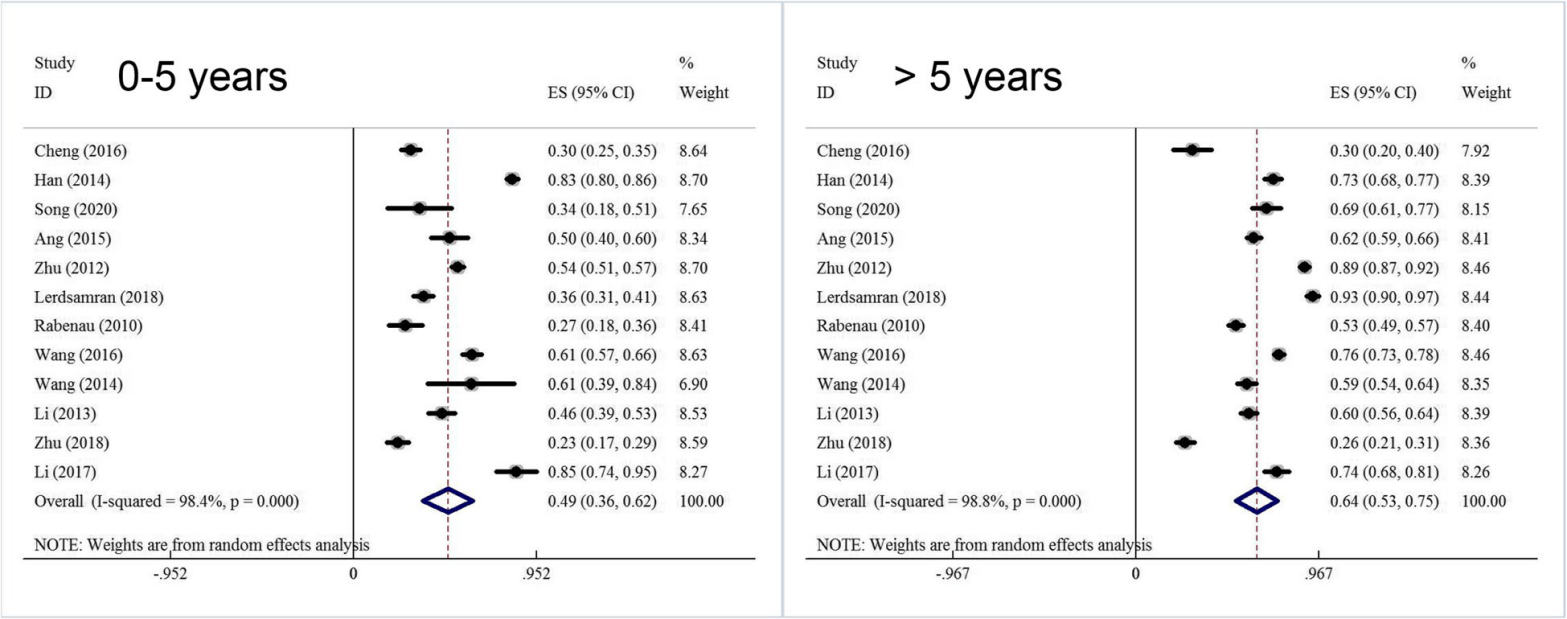
Forest plots for the seroprevalence of CoxA16 antibody among people in different age groups

Meta-analysis has been performed on the overall population and stratified based on different genders and age groups. A summary of the meta-analysis has been shown in Table 2. According to the heterogeneity test, significant heterogeneity was found among the studies, and the random-effect model has been used for meta-analysis. The results have showed that the seroprevalence of CoxA16 antibody was 56.3 % (95 %CI: 47.7 %~64.9 %) in the overall population and 55.1 % (95 %CI: 44.1 %~66.1 %) in the Chinese population (Fig. 2). Subgroup analysis by gender has revealed that the seroprevalence of CoxA16 antibody was 56.1 % (95 %CI: 45.2 %~67.1 %) in males and 60.0 % (95 %CI: 50.0 %~69.9 %) in females (Fig. S1 in supplemental materials). Subgroup analysis by age groups has revealed that the seroprevalence of CoxA16 antibody was 17.5 % (12.4 %~24.5 %) in the < 1 age group, 37.5 % (31.2 %~45.5 %) in the 1 ~ 3 age group, 50.8 % (41.6 %~60.0 %) in the 4 ~ 5 age group, 49.1 % (95 %CI: 36.2 %~62.0 %) in the 0 ~ 5 age group and 63.9 % (95 %CI: 53.1 %~74.7 %) in the over 5 age group (Fig. 3).

**Table 2:** Meta analysis results of the seroprevalence of CV-A16 (with 95 % confidence interval) in people by ethnicity, gender and age group

Group	Seroprevalence (%)	95 %CI (%)	Heterogeneity (*P*-value)	I^2^ (%)	Model	Begg’s (*P*-value)	Egger’s (*P*-value)
Ethnicity							
Overall	56.3	47.7 ~ 64.9	< 0.001	99.1	Random	0.228	0.105
Chinese	55.1	44.1 ~ 66.1	< 0.001	98.8	Random	0.335	0.156
Others	61.0	58.8 ~ 63.2	0.357	2.9	Fixed	0.224	0.135
Gender							
Male	56.1	45.2 ~ 67.1	< 0.001	98.0	Random	0.436	0.213
Female	60.0	50.0 ~ 69.9	< 0.001	97.4	Random	0.640	0.202
Age							
< 1 yrs	17.5	12.4 ~ 24.5	< 0.001	88.6	Random	0.336	0.215
1–3 yrs	37.5	31.2 ~ 45.5	< 0.001	92.3	Random	0.569	0.425
4−5yrs	50.8	41.6 ~ 60.0	< 0.001	87.6	Random	0.758	0.628
0 ~ 5 yrs	49.1	36.2 ~ 62.0	< 0.001	98.4	Random	0.837	0.266
> 5 yrs	63.9	53.1 ~ 74.7	< 0.001	98.8	Random	0.115	0.380

### Sensitivity analysis and publication bias

A sensitivity analysis has been carried out by calculating the pooled seroprevalence of CoxA16 antibody of the remaining studies after excluding one study at a time. The results of the sensitivity analysis have showed that the meta-analysis results were stable. In addition, there was no evidence of publication bias in the current meta-analysis according to the Begg’s funnel plots and Egger’s linear regression test, the results have been shown in Table 2 (Fig. S2 and S3 in supplemental materials).

## Discussion

CoxA16 virus is a common enterovirus that causes acute infection in children. It alternates or joins in prevalence with EV71 virus and is the main pathogen of HFMD. CoxA16 infection has become a major public health issue in the western pacific region due to its high transmission rate, especially in recent years [28]. Therefore, it is very important to carry out study on the determination of CoxA16 neutralizing antibody, so as to understand and predict the epidemic trend of HFMD and put forward prevention and control measures.

The present meta-analysis included 14 published studies containing a total of 9562 subjects, among which 5682 had positive neutralizing antibodies against CoxA16. The positive rate of CoxA16 neutralizing antibody in the serum of healthy people was relatively high with 56.3 % (95 %CI: 47.7 %~64.9 %) in the overall population and 55.1 % (95 %CI: 44.1 %~66.1 %) in the Chinese population. The meta-analysis also showed that the positive rate of CoxA16 neutralizing antibody was slightly higher in females than in males. This result was consistent with previous studies conducted in China, Singapore and Germany [17, 18, 21].

The meta-analysis also showed that the positive rate of CoxA16 antibody increased gradually from 1 to 5 years old and remained at a high level after 5 years old. Unfortunately, the positive rate of CoxA16 neutralizing antibodies was not analyzed for infants under 12 months of age. Studies have shown that with the natural attenuation of mother-to-child antibody, the antibody level of newborns decreased gradually from birth to the first year of life. A mother-newborn matching study in Jiangsu, China, found that the average maternal antibody positive rate at birth was 90 %, higher than EV-A71 infection level. However, the antibody level attenuated rapidly in infants of 0 ~ 12 months, which was similar to the change trend of EV-A71. After 1 year of age, antibody levels gradually increased with time due to increased exposure opportunities [19].

A retrospective study of the seroepidemiology of antibodies against EV71 and CoxA16 in prenatal women and their infants conducted by Mao et al. showed that the level of maternal antibody titers decreased dramatically during the first 7 months and remained at a relatively low level thereafter [16]. During the period of 4 ~ 6 years old, children began to study in kindergartens and live together with others, which may lead to increased exposure and infection opportunities of enterovirus and more likely to get and spread HFMD. After that period, children’s antibody level to enterovirus will gradually rise. Rabenau et al. found that the positive rate of CoxA16 neutralizing antibody in German children increased from 27 % in 1–4 years old to 52 % in 5–9 years old [21]. The present meta-analysis showed that CoxA16 antibody levels were significantly higher in children over 5 years of age than in children under 5 years old.

There are some limitations in this meta-analysis. Firstly, there was considerable heterogeneity in this meta-analysis, the causes may relate to the study area, the age range of the study population, the sampling time of objects in the studies and other factors. Due to the insufficient information provided in the original literature, subgroup analysis and meta-regression analysis cannot be carried out further. Secondly, the included literature was mainly from China, and the included regions are not evenly distributed, so the meta-analysis results may not reflect the positive rate of CoxA16 neutralizing antibody in the whole population. Finally, only English and Chinese literatures were included in this meta-analysis, so language bias may exist in this study.

## Conclusions

In summary, this meta-analysis showed that the proportion of CoxA16 virus infected population is relatively large, among which the CoxA16 positive rate is the lowest among people under 5 years old. The newborns’ level of CoxA16 neutralizing antibody from mothers is limited, and given there is no efficient vaccine specifically for this so far, the key to control the CoxA16 epidemic lays in prevention and control of children under 5 years old. In the future, public health departments should strengthen the publicity, education and prevention guidance on the prevention and control of HFMD, and urge parents to take personal protection for infants and young children.

## Supplementary Information

**Additional file 1: Figure S1.** Forest plots for the seroprevalence of CoxA16 antibody among people in different genders.

**Additional file 2: Figure S2.** Sensitive analysis for the seroprevalence of CoxA16 antibody among people in the overall population.

**Additional file 3: Figure S3.** Begg’s funnel plot for the seroprevalence of CoxA16 antibody among people in the overall population.

**Additional file 4: Table 1.** The search strategy of seroprevalence of CoxA16 antibody among people.

## Data Availability

The datasets used and/or analysed during the current study are available from the corresponding author on reasonable request.

## Abbreviations

HFMD: Hand foot and mouth disease
CoxA16: Coxsackie virus group A type 16
EV-71: Enterovirus 71
AHRQ: Agency for Healthcare Research and Quality
PRISMA: Preferred reporting items for systematic reviews and meta-analysis
95% CI: 95% confidence interval
