# Supplementary figures and images for: Seroprevalence of coxsackievirus A16 antibody among people of various age groups: a systematic review and meta-analysis
Source: Arch Public Health. 2021 Sep 17;79:166. doi: 10.1186/s13690-021-00688-z (PMC8447778; doi:10.1186/s13690-021-00688-z)

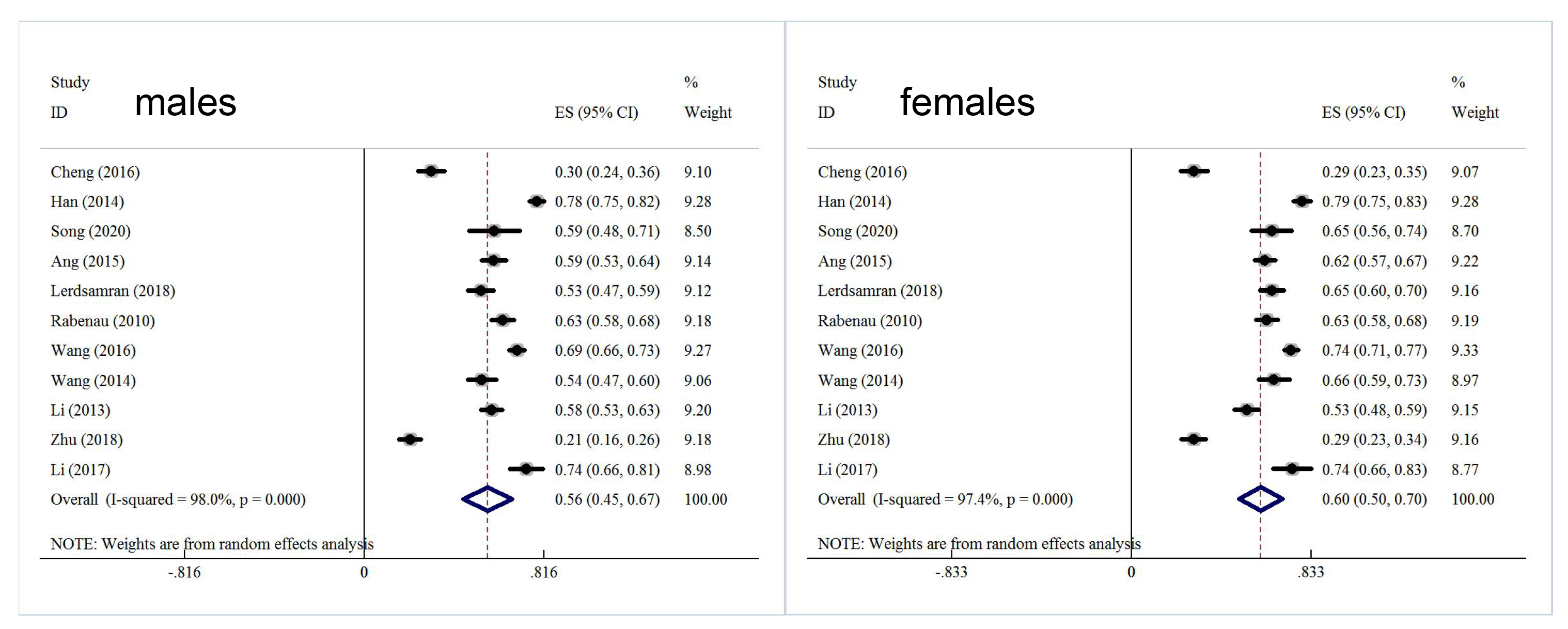

Supplement: Supplementary file 1 — Additional file 1: Figure S1. Forest plots for the seroprevalence of CoxA16 antibody among people in different genders. [file 13690_2021_688_MOESM1_ESM.tif]

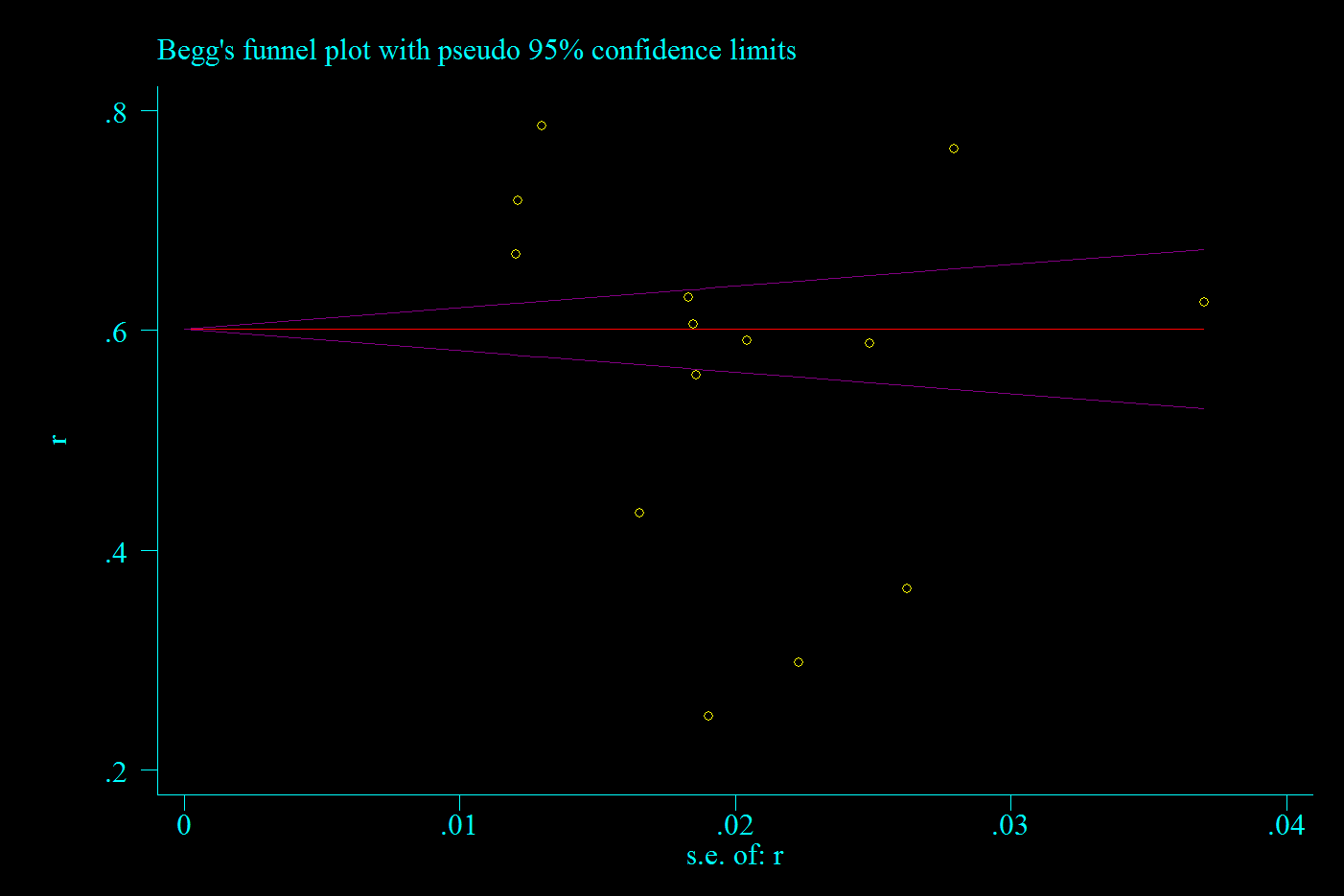

Supplement: Supplementary file 2 — Additional file 2: Figure S2. Sensitive analysis for the seroprevalence of CoxA16 antibody among people in the overall population. [file 13690_2021_688_MOESM2_ESM.tif]

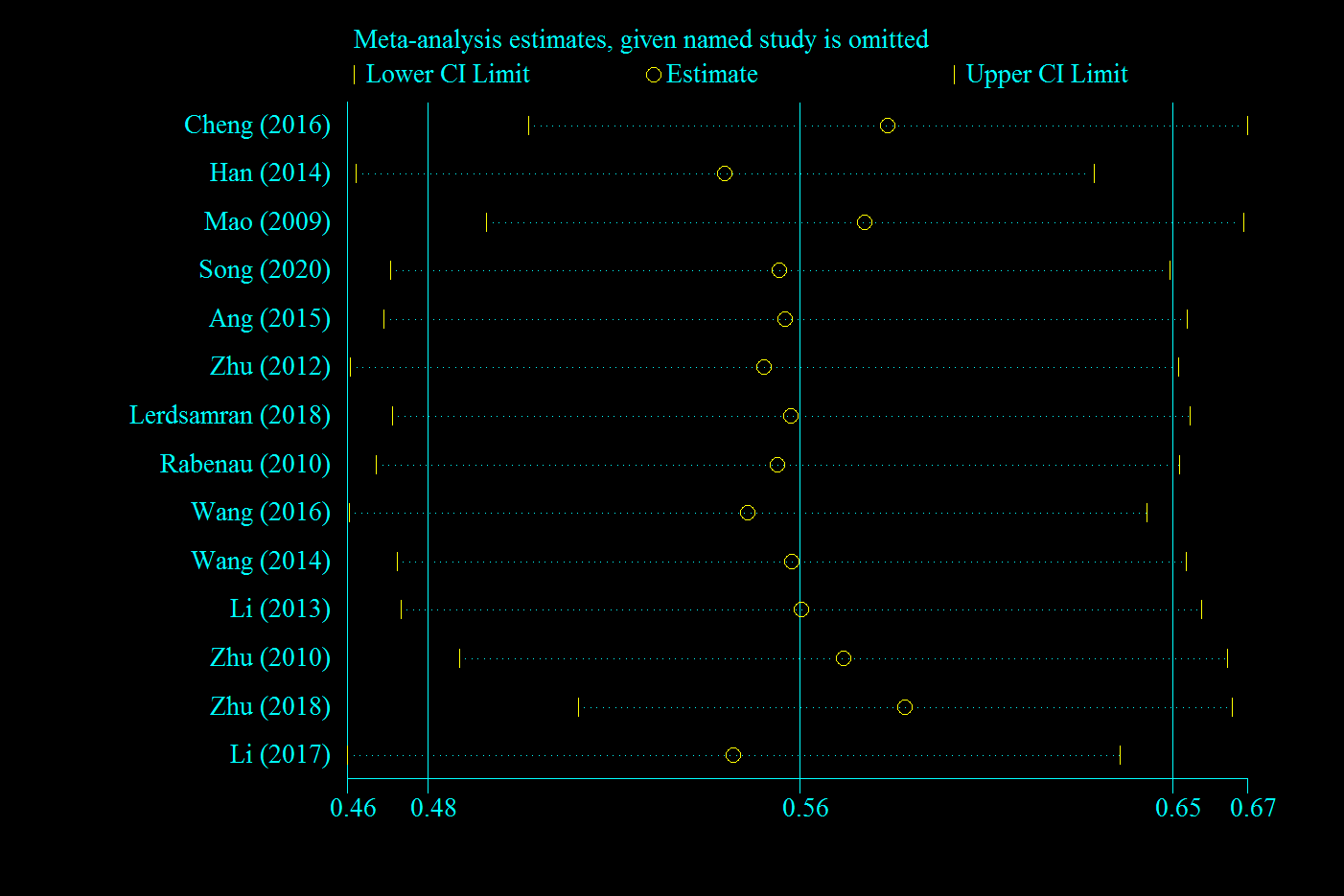

Supplement: Supplementary file 3 — Additional file 3: Figure S3. Begg’s funnel plot for the seroprevalence of CoxA16 antibody among people in the overall population. [file 13690_2021_688_MOESM3_ESM.tif]
