# Supplementary material for: Seroprevalence of coxsackievirus A16 antibody among people of various age groups: a systematic review and meta-analysis
Source: Arch Public Health. 2021 Sep 17;79:166. doi: 10.1186/s13690-021-00688-z (PMC8447778; doi:10.1186/s13690-021-00688-z)
Supplement: Supplementary file 4 — Additional file 4: Table 1. The search strategy of seroprevalence of CoxA16 antibody among people. [file 13690_2021_688_MOESM4_ESM.doc]

**Supplementary table 1.** The search strategy of seroprevalence of CoxA16 antibody among people.

| **Databases** | **Searches** |
| --- | --- |
| **China National Knowledge Infrastructure (CNKI)** | [(主题=手足口) OR (主题=柯萨奇) AND (主题=抗体) OR (主题=血清流行病)](https://kns.cnki.net/KNS8/AdvSearch?id=48&dbcode=SCDB&searchtype=gradeSearch&ishistory=1) |
| **WanFang Data** | 主题:(手足口) or 主题:(柯萨奇) and 主题:(血清流行病) or 主题:(抗体) |
| **Pubmed** | 1: (((((hand foot and mouth disease[All Fields])) OR (HFMD)) OR (coxsackievirus A16)) OR (CA16)) OR (CoxA16)  2: (((((((((seroprevalence[All Fields])) OR (seroprevalent)) OR (seronegative)) OR (seropositive)) OR (seroepidemiology)) OR (seroepidemiological)) OR (serologic)) OR (serological)) OR (antibody)  #1 and #2 |
| **Embase** | (“hand foot and mouth disease” OR “HFMD” OR “coxsackievirus A16” OR “CA16” OR “CoxA16”) AND (“seroprevalence” OR “seroprevalent” OR “seronegative” OR “seropositive” OR “seroepidemiology” OR “seroepidemiological” OR “serologic” OR “serological” OR “antibody”) |
| **Cochrane Library** | (“hand foot and mouth disease” OR “HFMD” OR “coxsackievirus A16” OR “CA16” OR “CoxA16”) AND (“seroprevalence” OR “seroprevalent” OR “seronegative” OR “seropositive” OR “seroepidemiology” OR “seroepidemiological” OR “serologic” OR “serological” OR “antibody”) |
